# Monitoring bands during the Norwegian national day parade: a case study on urban distributed acoustic sensing

**DOI:** 10.1038/s41598-025-97017-z

**Published:** 2025-04-20

**Authors:** Robin Andre Rørstadbotnen, Jo Eidsvik, Jan Langhammer, Martin Landrø, Osman Mohammad Ibrahim

**Affiliations:** 1 Acoustic group, Department of Electronic Systems, Norwegian University of Science and Technology (NTNU), O. S. Bragstads Plass 2A, 7491 Trondheim, Norway; 2 Centre for Geophysical Forecasting, Norwegian University of Science and Technology (NTNU), O. S. Bragstads Plass 2A, 7491 Trondheim, Norway; 3 Department of Mathematical Sciences, Norwegian University of Science and Technology (NTNU), Alfred Getz’ vei 1, 7491 Trondheim, Norway; 4 City of Oslo, Emergency Planning Agency, Olav Vs gate 4, 0037 Oslo, Norway; 5 Present Address: Sensnet Analytics AS, Borgundvegen 340, 6009 Aalesund, Norway

**Keywords:** Distributed acoustic sensing, Fiber optic sensing, Urban monitoring, Norwegian national day, Crowd characterization, Walking frequency, Applied optics, Acoustics, Geophysics

## Abstract

Existing networks of fiber optic telecommunication infrastructure can be used to measure acoustic events. For this purpose, a laser instrumentation is attached to a “dark fiber” turning it into a Distributed Acoustic Sensing (DAS) device. In May 2023, a DAS test was conducted to measure acoustic activity in Oslo, Norway. The main purpose was to measure the “pulse” of the Oslo city center during the Norwegian National Day parade. Additionally, five days before and after the National Day were recorded for reference to daily acoustic background noise conditions in Oslo. Data during the National Day captured the yearly parade in which schools and bands participate. Using this data, it was possible to detect the participating bands, analyze their frequency content, and estimate their walking speed and step length. High-order harmonics were recognized in the frequency response for the bands. A total of 88 bands participated in the parade and 87 were detected using the harmonic characteristics. While one individual band could be tracked before the main parade over separate streets, it was challenging to continue the track for other bands within the parade. The test revealed that DAS can be used as part of decision support systems for crowd monitoring.

## Introduction

Motivated by applications in urban monitoring, a Distributed Acoustic Sensing (DAS) dataset from Oslo, Norway, is presented and analyzed. DAS has shown great potential in urban traffic monitoring which could be highly useful for transportation decision support systems^1,2^. The urban DAS dataset from Oslo is used to go beyond this topic to showcase how the sensing technology is useful to detect, track, and understand marching band movement within the Norwegian National Day parade. This can be valuable in urban crowd management situations involving community services, evacuation operations, and police force allocation. In such situations, DAS can complement other data sources such as airborne sensing data, camera information, cell phone positioning systems, or wifi connection data. DAS can further work as an independent source when other data types are not applicable, say, because of light conditions, connection issues, or privacy concerns. For instance, DAS is not affected by weather conditions (like fog or heavy rain) that might render footage from security cameras useless due to limited visibility. DAS has already been successfully applied to perimeter security to detect intrusion along a railway^3^ and to classify signals from humans, animals, and vehicles^4^.

DAS technology has become increasingly popular in the last decade because of its promising data and research outputs^5–7^. It is, however, still an emerging technology that needs further exploration to fully understand its potentials and limitations within different scientific branches. In existing telecommunication fibers, there are small density fluctuations that act as scatterers to the laser light sent into the cable from the instrumentation. Hence, a small portion of the laser light is reflected back to the instrumentation. Because the positions of the scatterers are affected by compression and stretching of the fiber, the backscattered portion of the light will undergo a phase change. This measured phase change of the laser light is a function of the strength of the acoustic force. The fiber is synthesized to a series of receiver points that can be spaced from tens of centimeters to tens of meters, depending on the application and Interrogator Unit (IU) used. The existing DAS technology has a great potential for re-purposing the existing fiber optic network infrastructure into distributed sensor systems for monitoring. There are millions of km of fiber cables already laid out for telecommunication purposes that can be effectively re-purposed and hence increase the receiver coverage worldwide^8^. Moreover, it is possible to employ tailor-made solutions, i.e., install dedicated fibers in areas of interest, e.g., along critical infrastructure like pipelines, bridges, or rail lines. DAS has already been used in a broad range of applications, including sub-sea applications like whale tracking^9,10^, oceanographic analysis^11^, geophysical studies^5^, environmental monitoring^12^ and urban applications^1,2,13,14^. The rapidly growing literature on DAS demonstrates its potential for generating new methods, insights, analysis, and monitoring systems.

Research on crowd monitoring using DAS is still in its infancy, and there are few studies on the topic. Jakkampudi et al.^15^ observe the footsteps of individual pedestrians on an urban fiber optic array and use a convolutional neural network for a large-scale footstep detection study. Zhou et al.^16^ show that DAS and machine learning can be combined for footstep recognition, benchmarking DAS performance against geophone receivers. As for the current study, an urban DAS dataset was acquired using an existing telecommunication network to further explore the topic of crowd monitoring. The dataset was acquired before, during and after the Norwegian National Day (May 17) in 2023. The benefit of acquiring DAS data on May 17 is that most public transport is halted while the parade takes place. Hence, the dominant signal is generated by the crowd. To capture the daily life acoustic background noise condition in Oslo, a five-day period was recorded before and after the National Day, resulting in 11 days of data.

On May 17, school classes and marching bands walk in a parade along a preplanned path through the Oslo city center. For this study, a marching band is defined as a group of 10–50 persons playing instruments while marching. Marching bands are henceforth referred to as bands. A fiber route that coincide with parts of the parade path was patched to record and characterize the schools and bands. By recording throughout the event, differences in signatures were obtained. From the DAS data it was possible to detect, monitor and track crowds within the 17th of May parade. Results indicate different frequency characteristics between the schools and the bands. This illustrates the potential of DAS on an existing telecommunication network for crowd monitoring. The paper by Wang et al.^17^ contains similarities with the current study. The authors investigated the DAS response of the Rose Parade in California and discuss the signal characteristics of cars and bands within that parade. Moreover, they point out the difference in frequency content related to higher order harmonics for the bands. In the current paper, 87 different bands are successfully detected and analyzed for tracking, frequency response estimation and step lengths characterization over the duration of the parade.

The paper is organized as follows. Section “Distributed acoustic sensing data” outlines the DAS data acquisition, background urban events detection on the fiber, and basic information about the May 17 parade. Section “Analysis of parades observed on DAS data” describes the data analysis, including the description of a load model for uncoordinated pedestrians, a method for estimating the speed of the parade and an approach for tracking the parade. Section “Results and discussion” presents and discusses main results from the parade analysis.

## Distributed acoustic sensing data

### Data acquisition

**Fig. 1:**
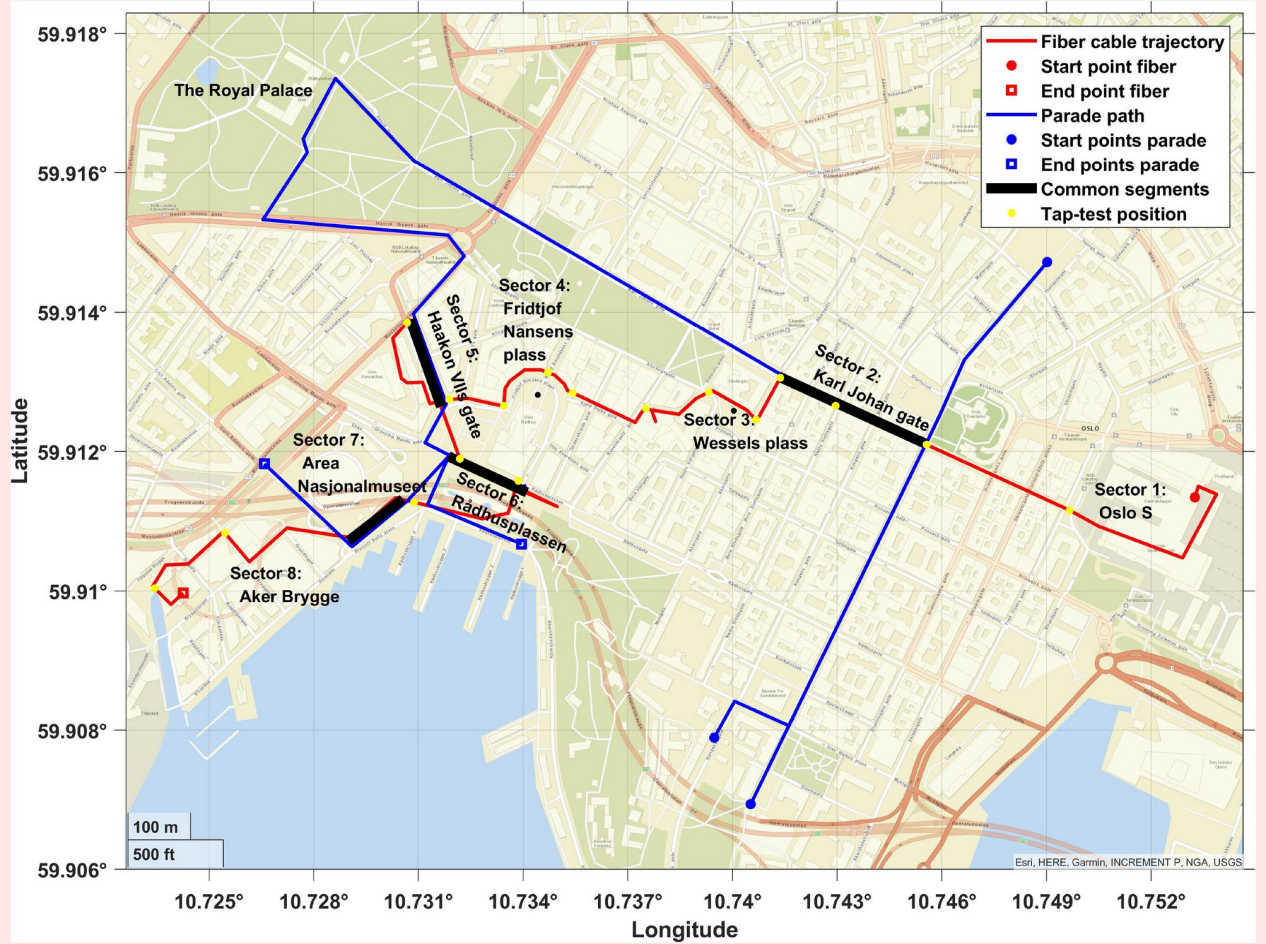
Fiber cable lay-out and parade path. Map of the fiber cable trajectory (red), the path of the National day parade (blue), and common segments where the parade moves parallel to the fiber cable (black). The red asterisk indicates the start point of the fiber cable trajectory (the IU location) and the blue asterisks show where the parade path starts. The respective squares indicate the endpoints. The background map was created using the ESRI world street basemap in MATLAB^18,19^.

A single “dark fiber” within the commercial fiber optic communication network in Oslo was made available for the acquisition period. Patches were made to connect different fiber paths, forming a continuous line traversing a selected portion of the Oslo City center (see Fig. 1). To enhance readability, streets and areas the fiber cross are referred to as ’sectors’. See Fig. 1 for their definitions. The OptoDAS IU was used to turn a 3.88 km long fiber cable into a discrete seismic sensor array with receivers every 1.02 m. This channel spacing was sufficiently small to avoid spatial aliasing (see Supplemental Section S1 online). The fiber was continuously interrogated from May 11 to May 22, 2023, with a temporal resolution of 2500 Hz and a gauge length of 2.04 m. The DAS instrumentation was controlled and monitored remotely by a standard 4G cellular network, which made it possible to adjust and download acquisition parameters and diagnostic displays in near-real time. The data quality of the IU is dependent on the sweep duration of the laser signal. A poorly chosen value will introduce extra instrument noise after high-amplitude events (crosstalk; see Section “Urban events detected using fiber optic sensing” for more details). The parameters were adjusted to visually find the best Signal-to-Noise Ratio (SNR) of such high-amplitude events. The sweep duration varies depending on the temporal resolution. For the 2500 Hz sample rate used, the sweep duration that gave the best data quality was found to be 50 µs. This sweep duration was kept throughout the study period.

To extract strain signals related to urban activities, four preprocessing steps were carried out. (1) The data was converted from time-differentiated phase change (as stored by the IU during acquisition) to strain. (2) The data was resampled according to the highest frequency of interest. Specifically, the data was downsampled by a factor of 40, i.e., decimated to a new sample rate of 62.5 Hz. A low-pass filter with a cut-off frequency of 26 Hz was applied to avoid temporal aliasing. (3) The data was detrended and a demean was applied. (4) A Tukey window and a 4th order Butterworth high-pass filter with a cut-off frequency of 1 Hz were applied.

A tap test was performed on the installation day to correlate the positions of the fiber channels with latitude and longitude coordinates of physical locations. A hammer was used as it generated signals strong enough to be recorded on the portion of fiber closest to the blow. The test was carried out by tapping the sidewalk directly above the fiber at the start and end point of straight portions of streets in which the fiber traversed (see Fig. 1 for tap positions). If a street was straight, the start and end was tapped, if the street was curved or change direction tapping was carried out more frequently, e.g., at turning points. The hammer blows were carried out by one individual which aimed to hit the pavement with the same force at every location. In some locations the fiber had poor coupling and more force was needed to observe the signal. Another individual examined the live data feed and gave feedback to the hammer operator if more force was required to observe it in the data. The apex of the generated signal was picked as the channel associated with the geographical coordinates. Note that the fiber was located at unknown depths underneath the sidewalk. It was therefore not possible to directly tap the fiber cable. Hence the need for a hammer (or similar sources).

### Urban events detected using fiber optic sensing

**Fig. 2:**
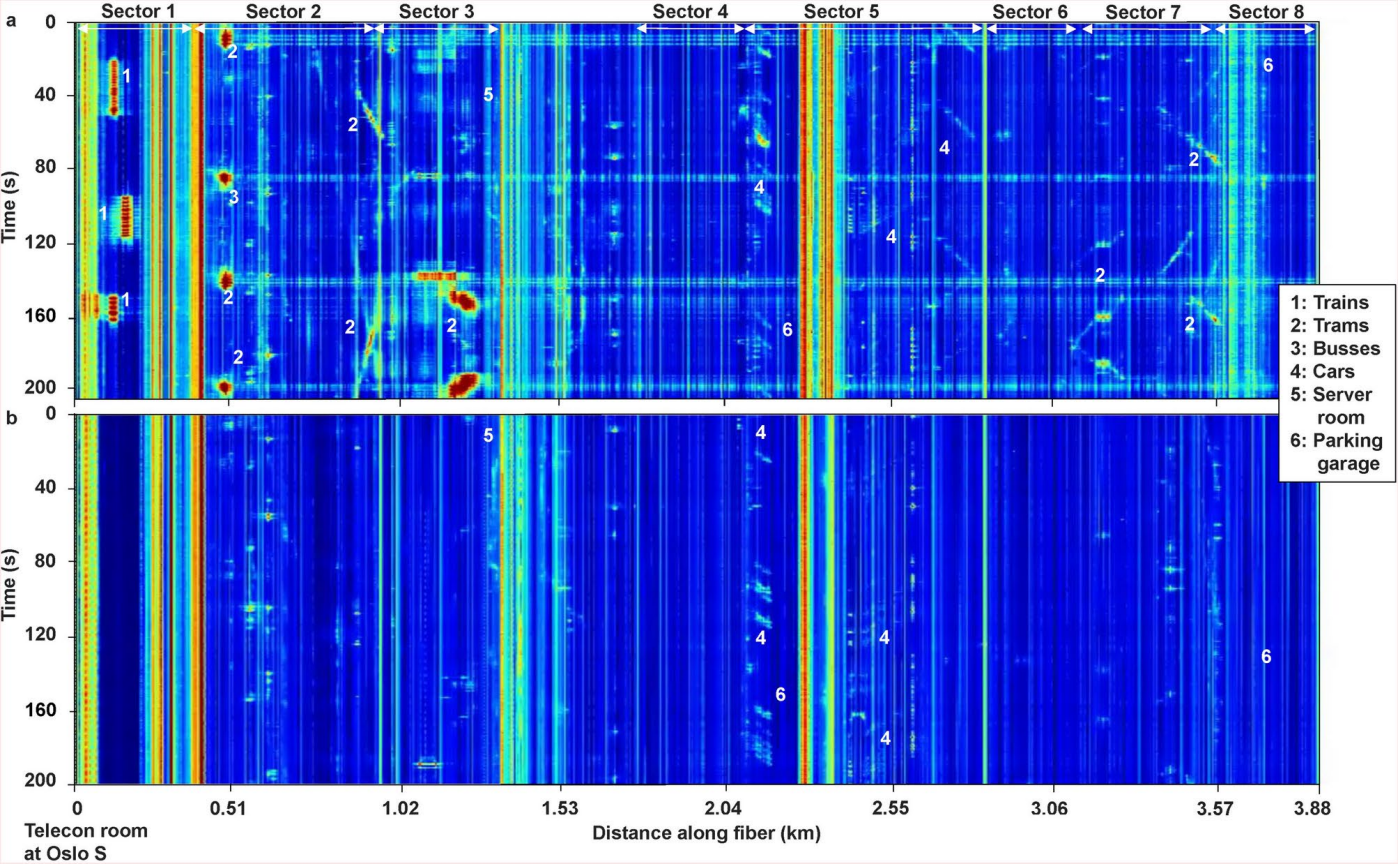
Typical DAS background noise data for day and night. (**a**) Waterfall plot from 2023.05.16 starting at 05:53:33 (UTC). (**b**) Waterfall plot measured at night on 2023.05.14 starting at 01:27:30 (UTC). Compared to (**a**) there is significantly less activity during night (**b**). Some key responses and spatial locations of public transportation and cars in various directions over and along the fiber cable are annotated in both (**a**) and (**b**). These annotations are presented and discussed in the main text Section “Urban events detected using fiber optic sensing”.

Figure 2 shows waterfall plot examples for day and night. The display in Fig. 2a is extracted from a usual period of weekday rush hour in Oslo. During this time period people are traveling to work and an increase in, e.g., public transport can be observed. The data starts Monday 2023.05.16 05:53 (UTC). The most obvious events are various kinds of public transportation. At the main train station (Sector 1) and the roads outside the train station, the fiber is perpendicular to the direction of rail tracks and roads (Fig. 1). The data signature for public transport for such cases is a dot stretched in time (vertical axis in Fig. 2) rather than a moving object that can be followed at different distances (horizontal axis in Fig. 2) along the fiber. Such events are most pronounced on the first 500 m of the fiber (annotations 1, 2, and 3 in Fig. 2a). For trains arriving in Sector 1 (annotation 1 in Fig. 2), it is possible to detect which track the various trains arrive on. The tracks are identified by signals along channels with SNR above a threshold value. By manual inspection, the SNR threshold was set to four as most of the trains were observed to exceed this threshold. As the fiber is perpendicular to the train tracks and the tracks are separated by more than two gauge lengths it is possible to observe which channel is associated with each track due to the clear move-out between the different arriving trains. Furthermore, by extracting all instances where signals exceed the SNR threshold, it is possible to automatically count the number of trains arriving on each track and characterize the number of carts on each train. In fact (at distances 50 to 200 m, Fig. 2a), individual events are observed and related to each cart of the trains entering the station.

Cars measured at places where the fiber goes parallel to the main traffic direction are annotated as  4 in Fig. 2. Heavy traffic can produce very strong fiber vibrations, and the laser pulse polarization is modified when passing the point of strong vibration. This results in crosstalk when the modulated pulse is used to interrogate subsequent sensor points. The crosstalk is observed as horizontal lines (lines along the distance axis). This is particularly clear when trams cross a section where the fiber runs underneath the tram rails (annotations 2, 3 in Fig. 2a).

Figure 2 also show vertical stripes (annotations 5, 6), for instance at 0.40 km, 1.30 km, 2.30 km, and 3.58 km, which are either caused by the fiber entering a server room with constant noise from fans or a parking garage with industrial fans or pumps near the fiber. The vertical stripe at 1.30 km is due to the fiber being inside a server room (information from the owner of the fiber cable, Telia Norge AS), whereas the stripes at 2.30 km and 3.15 km are due to fans or pumps in parking garages.

The acoustic impact during the city rush hour can be compared to a quiet period during the middle of the night. Fig. 2b shows the background noise for such a period (starting Sunday 2023.05.14 01:27 UTC). The most pronounced difference is the reduced noise from vehicles, trams and trains. Semi-constant and stationary noise from underground tunnels, garages, and buildings, are still visible. The fan or pump amplitudes at 2.30 km decrease substantially from day to night, while the signal at 3.15 km disappears.

### Observations of the May 17 parade in Oslo City

The main rationale for choosing this time period to perform measurements in the city center of Oslo was to enable DAS recording during the Norwegian National Day (May 17). At this day most of the public transportation is halted over a few hours, allowing DAS recording of the impact of the largest possible differences between moving crowds of people relative to the monitoring of daily-life acoustic noise in a city center. When the heavy urban traffic is paused, the larger crowd of people will be the most prominent signal generator in specific areas in the city. Oslo is known for its children’s school and band parade on the national day. As this parade moves in a planned path its signature recorded on the DAS data can easily be studied. The path of the parade is depicted as blue lines and the DAS trajectory as red lines in Fig. 1.

**Fig. 3:**
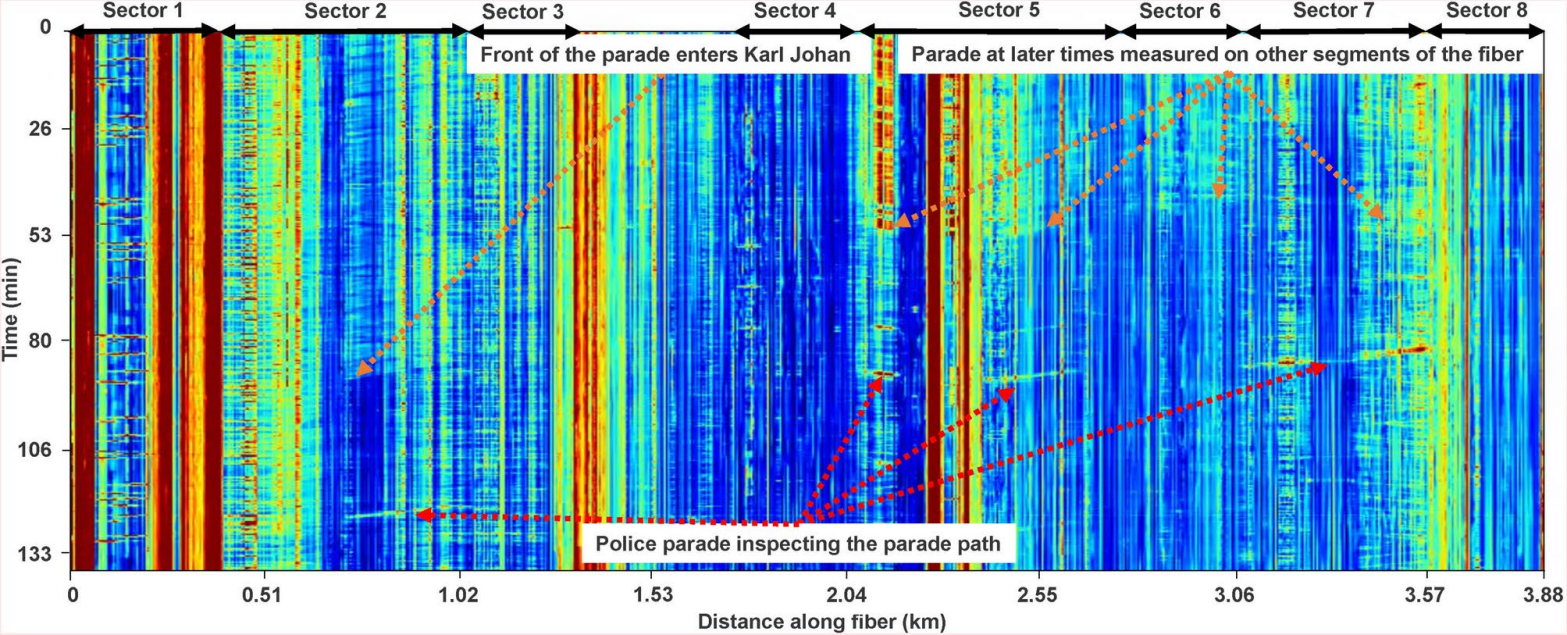
DAS parade response. Waterfall plot showing the impact of the parade. Note the change in the *y*-axis showing (min) compared to Fig. 2. The signal generated by the parade is measured at different portions of the fiber as the crowd moves from Sector 2 (Karl Johan Street) to other sectors along the fiber (see Fig. 1).

A waterfall plot of more than 2 h before and during the time of the parade is shown in Fig. 3. As the available fiber path only covers parts of the parade trajectory, it is observed at specific portions of the fiber (black bold lines in Fig. 1). During the period depicted in Fig. 3, the parade could be observed as it moved up Sector 2 (Karl Johan street). Later, the front of the parade is moving down Sector 5 (Haakon VIIs street), while a trail of the parade is still moving up Sector 2. This illustrates the possibility of following the movement and characterizing the different parts of the parade simultaneously.

## Analysis of parades observed on DAS data

In this section the key methodological elements used to analyze the DAS data containing the May 17 parade are described. First, vertical forcing of pedestrians is analyzed via the frequency characteristics in the DAS data. Then, the walking pace of the parade is assessed by regression analysis and signals are tracked by applying crosscorrelation over multiple DAS channels.

### Load model for uncoordinated pedestrians

There exist models for the frequency response of pedestrians^20,21^. One such model is for the vertical force, $F_1(t)$, over time *t*, induced by a group of uncoordinated pedestrians:

1 $$\begin{aligned} F_1(t)=W\left[ 1 + \sum _{n=1}^N \sqrt{N} \alpha _n \sin {(n2\pi f_p t + \phi _n)}\right] . \end{aligned}$$

Here, *W* is the weight of the pedestrians; *N* the number of harmonics included in the model; $\alpha _n$ the dynamic loading factor; $f_p$ the pacing frequency or dominant mode, and $\phi _n$ the phase angle for the *n*th load harmonic.

To compare the DAS response to the established load model in Eq. (1), short-term Fourier transforms and spectral analysis are applied to the DAS data. The frequency content of the data is extracted from spectrograms estimated for different channels along segments of interest. All spectrograms presented in this paper are calculated using a 4096 sample Hann window, with 90% overlap, providing a temporal resolution of 6.56 s and a frequency resolution of 0.15 Hz. To extract the frequencies a window is constructed around the first harmonic mode (1.5–2 Hz). This window is introduced to avoid picking frequencies related to higher modes (above 2 Hz) or energy from other low-frequent acoustic sources (below 1.5 Hz). Within the frequency window, the frequency component corresponding to the maximum energy for each time step is found.

The first harmonic mode frequencies are in line with the theoretical model in Eq. (1) and controlled analysis of pacing speed^20,22^. The higher-order harmonics are multiples of the first mode. When a band is walking in step, there are pronounced higher-order harmonics, especially clear for the trained bands. For an ordinary school class somewhat out of step, the higher-order harmonics are weaker.

For the analysis of the DAS data, an extension of Eq. (1), as suggested by Brownjohn et al.^21^, was used. This model assumes that the pacing frequency is a random variable with a Gaussian density: $F_p \sim N(f_p, \sigma _{f_p}^2 )$, to incorporate the uncertainty in the multi-pedestrian harmonic frequencies. To account for the random frequency, a version of approximate Bayesian computing was employed^23^. This involves using Monte Carlo sampling over independent prior distributions for the mean dominant frequency ($f_p$), the frequency standard deviation $\sigma _{f_p}$ and the first four dynamic loading factors $\alpha _n$ ($n=1,2,3,4$). Assuming independent contributions from $N=10$ pedestrians, each Monte Carlo sample is plugged into Eq. (1). Prior samples are accepted if the mismatch in data spectral density and modeled spectral density is smaller than a threshold. Aiming for low overall mismatch as well as sharp modes near dominating frequencies, prior samples are accepted giving an integrated mismatch of less than 70 in the sum of mean absolute difference and 100 in the mean square difference.

### Walking speed and track of the parade

For a more accurate analysis of the parade characteristics, it is tracked over time. By computing the speed of the parade, functional estimates over time can be stacked to improve SNR levels. It further allows a more detailed analysis of the variability in bands and schools walking in the parade.

The speed of the parade can be estimated using various techniques. Over a long distance and time-span, the walking speed will vary, but perhaps not so much that it influences the analysis in short time-space windows. First, the speed is estimated by picking the maximum amplitude of the moving front and using the associated offset and time values in a linear regression to find the slope. An iterative reweighted least squares method is used as it is more robust to outliers in the data^24^. The approach fits the mean and variance for the slowness *s*. This defines a Gaussian approximation for slowness, and Monte Carlo samples are generated from this distribution. Next, the relation $v=1/s$ is applied to each of these realizations, providing Monte Carlo samples of the walking pace in the parade.

Going beyond regression fitting of the speed, parts of the parade are tracked over time using crosscorrelation of their DAS signal characteristics. A group of pedestrians can be tracked if they produce clear signals in either the time or frequency domain. In this study, clear frequency modes for the different portions of the parade are observed. Therefore, crosscorrelation is conducted in the frequency domain. A channel with clear frequency modes is chosen as the stationary reference signal and it is crosscorrelated with subsequent channels along the segments of interest and over the duration of the parade. Denoting the reference spectral density at center time *t* and channel *x* by *r*(*f*, *t*, *x*), the crosscorrelation can be written as

2 $$\begin{aligned} (g\star r)_{t,x}[\tau ,y] = \int _{f_0}^{f_0+F} \overline{g(f,\tau ,y)}r(f,t,x)df, \end{aligned}$$

where $f_0$ is the lowest frequency, set below the first mode, and $f_0+F$ is the frequency of the highest mode observed ($\sim$10 Hz in this study). Profile $g(f,\tau ,y)$ is computed for all channels (*y*) and for all time periods ($\tau$) covering the duration of the parade. For each correlated channel, the zero-lag is extracted (see Supplemental Section S2 online). For example, when the autocorrelation is carried out the crosscorrelation coefficient will be 1 at the zero-lag which defines the start of the tracking. The subsequent channels will show a shifted maximum correlation coefficient relative to that of the autocorrelation. By following the maximum coefficient over the various channels a track is produced for the group of pedestrian chosen. Note that by performing crosscorrelation over the whole duration of the parade, the similarity (in terms of walking frequency) of the various portions of the parade can be assessed at the same time as the track.

**Fig. 4:**
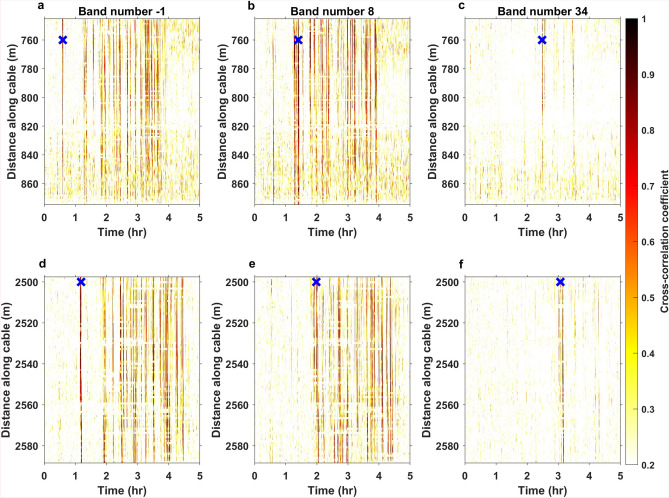
Band tracking using crosscorrelation. Crosscorrelation of bands -1, 8, and 34 using a frequency profile between 1 and 10 Hz (containing modes 1 through 4). The channel at 760 m is correlated with the other channels along Sector 2 (**a**–**c**) and Sector 5 (**d**–**f**). (**a**, **d**) Track of the police parade (band -1) before the actual parade starts. The track illustrates that the parade can be consistently tracked through both streets from one base trace. (**b**, **e**) Track examples containing many bands with similar frequency content. Hence, many lines with high crosscorrelation coefficients are obtained. (**c**, **f**) Track for a low-frequent band, dissimilar to the other bands. Hence, there are few lines in the displays. The blue cross in (**a**–**c**) indicates the time of the base profile in (**d**–**f**) the time is shifted according to the known walk time to Sector 5 from the base profile.

This approach of crosscorrelation is illustrated using selected reference bands over the duration of the parade for channels along Sector 2 and Sector 5. Figure 4 shows three different track examples for reference bands -1, 8, and 34. According to Oslo municipality^25^ 88 bands participated in the parade (denoted as bands 1 through 88). Additionally, two bands walking before the parade started have been identified, which were not part of the list (denoted as band -1 and band -2). Band -1 is trained and show clear modes. This band was successfully tracked along Sector 2, and could further be identified when it enters Sector 5, 35 min after exiting Sector 2. The blue cross in Fig. 4a shows the timing of the stationary trace, while the cross in Fig. 4d shows a shifted version representing the time it takes for the band to march from the stationary trace at 760 m to the first high-quality channel at Sector 5 (channel 2500 m from the IU). Fig. 4b, e show a second example where several bands with similar frequency characteristics march close to the band investigated (band 8). By zooming in around the predicted arrival at Sector 5 it is still possible to identify when the bands with similar frequency characteristics arrive. Although there is separation in time between these bands it is difficult to identify which track corresponds to band 8, due to the similar walking frequencies. The final example in Fig. 4c, f, shows band 34 which changes walking frequency as it walks along Sector 2 and Sector 5. Due to this change, the crosscorrelation coefficient decreases faster than for the other examples. It is, however, fewer large coefficient tracks produced as the walking frequency differ from the majority of the other bands. For the predicted arrival at Sector 5, band 34 has the same walking frequency as when it entered Sector 2. However, the band continues to change walking frequency, and the coefficient decreases along the profile.

Despite small variations, crosscorrelation and regression analysis of the parade walking pace gave similar results. Both indicate a pace of about 1 m/s.

**Fig. 5:**
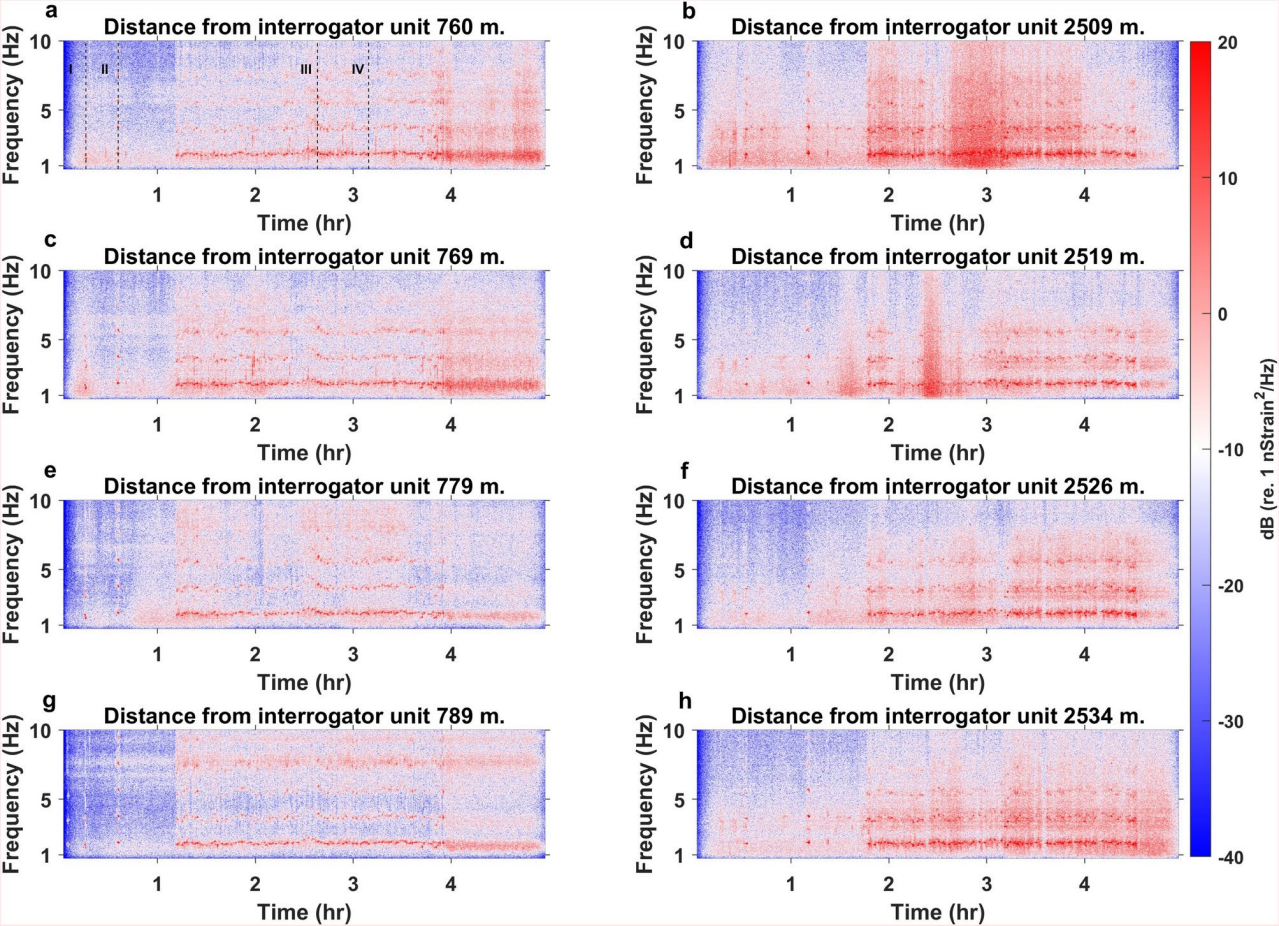
Frequency characteristics of the parade. Spectrogram representation of four traces along Sector 2 (left panels) and four along Sector 5 (right panels). Red colors represent the main frequency components in the DAS signals. For Sector 2, four to five modes can be observed for roughly 2.8 h, whereas Sector 5 contains three to four modes over a similar period. The modes are interpreted to be generated by the steps from groups of people walking in the parade. Annotations in (**a**) indicate the timing for events I, II, III, and IV to be presented later in Fig. 6.

## Results and discussion

### Characteristics of bands and schools

The analysis of the May 17 parade was done using DAS data recorded along Sector 2 and 5. The orange arrows in Fig. 3 highlight the front of the parade as recorded in both sectors, generated by the band marching in the front of the parade. By extracting the maximum amplitude of the signal the walking speed of the parade is computed using robust linear regression (Section “Walking speed and track of the parade”). The estimated walking pace along Sector 2 is $0.99\pm 0.06$ m/s and $1.15\pm 0.08$ m/s along Sector 5 (interval corresponding to the standard error). The higher walking pace along Sector 5 is attributed to the parade breaking up and moving faster once walking by the Royal Palace (see Fig. 1). For the sake of completeness, additional velocities for five clear linear events along Sector 2 are estimated to investigate changes in speed over the parade duration. The obtained average speed is 0.92±0.17 m/s.

Figure 5 shows the spectrogram of the parade signals recorded by four channels along Sector 2 and four along Sector 5. The signal energy increases substantially when the parade enters the streets at the 1.1 h mark for Sector 2 (left column) and the 1.8 h mark for Sector 5 (right column). When the parade moves along the fiber, 4 and 5 modes are observed. The fundamental mode frequency of the bands fluctuates around 1.8 Hz with varying intensity. This frequency range agrees with previously reported^26^ and modeled values^20^, as well as the characteristics observed in the Rose Parade^17^. Moreover, the over-harmonic frequencies are multiples of the fundamental mode frequencies, and over time they oscillate in the same manner with decreasing intensity as a function of harmonic number.

**Fig. 6:**
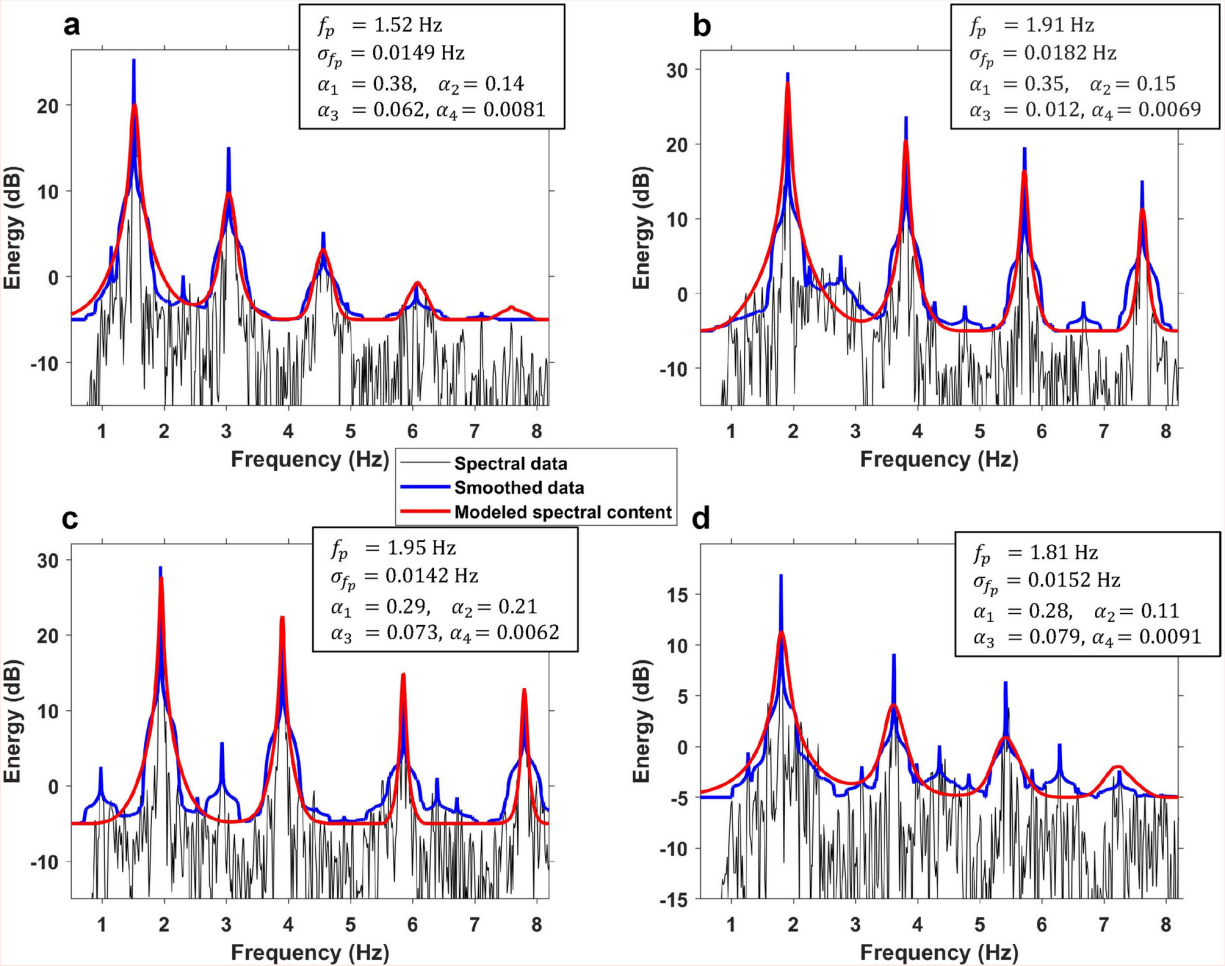
Modeled and observed multi-pedestrian walk frequencies. Modeled curves (red) for multi-pedestrian walk frequency amplitude spectra versus observed frequency amplitude spectra from DAS (black is un-smoothed and blue is smoothed). The parameters used to find the modeled curves are given in the top right corner for each subplot. (**a**) From event I (see Fig. 5a), (**b**) event II, (**c**) event III, and (**d**) event IV.

The difference between un-synchronized and synchronized motion is illustrated by frequency amplitude spectra in Fig. 6. These spectra are extracted for selected bands and schools (marked I–IV in Fig. 5a) recorded on channel 760 m from the IU. To quantify the model parameters matching the observed data, approximate Bayesian computing described in Section “Load model for uncoordinated pedestrians” is employed. Both observed and modeled spectra are given in Fig. 6 together with the estimated parameters. Figure 6a shows a band walking with low frequency and weak over-harmonics. Figure 6b, c depicts the frequency content of two different time samples with high amplitude arrivals, corresponding to synchronized bands. Conversely, Fig. 6d shows the frequency content for an un-synchronized school class (between two bands).

### Detection of bands

**Fig. 7:**
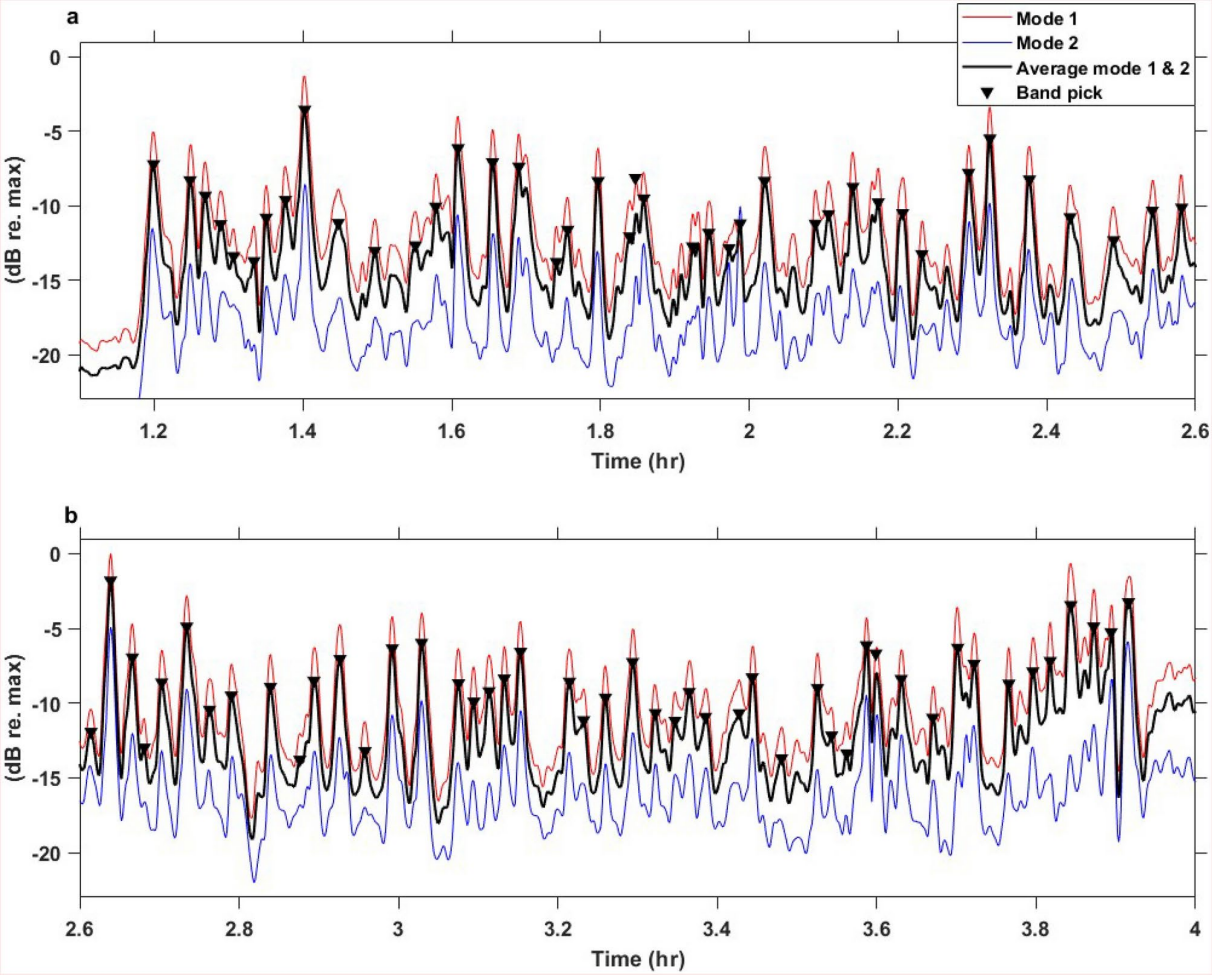
Stacked spectrogram profiles for all channels along a portion of Sector 2. The profile has been divided in two for a better illustration of the various peaks along the profile. (**a**) Contain the initial 1.5 h of the parade and (**b**) the final 1.3 h. The number of peaks equal to 87, which is one less than the reported number of bands in the parade. The three profiles depicted are from mode 1 (red), mode 2 (blue), and the average between modes 1 and 2 (black). The black triangles are automatically picked from the averaged profile.

Oslo municipality^25^ provides a list of schools and bands participating in the May 17 parade every year. According to this list, there were 110 schools and 88 bands participating in 2023. By using the DAS data recorded along Sector 2 these bands were attempted detected. This was done by computing spectrogram profiles for channels at distances 760 to 800 m from the IU to estimate profiles for modes 1 and 2. The channel range was chosen due to its high SNR. The spectrogram profiles were computed by summing frequencies over a specified interval enclosing the mode. For mode 1 the interval was 1.3–2.5 Hz, while it was 3.2–4.8 Hz for mode 2. The profiles are subsequently aligned and stacked to obtain the best possible profile, assuming a straight segment and using the obtained parade pacing speed. This resulted in the profiles depicted in Fig. 7. The performance of the stacking was tested using band -1 and resulted in a SNR that improved by a factor of 1.54 compared to the SNR of using only channel 760 m from the IU (see Supplemental Section S3 online).

Each band generates a clear fundamental mode and first over-harmonic (Fig. 5). After stacking the frequency interval enclosing these modes the bands are observed as peaks in the profile (Fig. 7) and can be extracted automatically. The number of peaks corresponds to the number of detected bands. To further enhance the peaks the average between the fundamental mode and the first-harmonic is calculated. The peaks in the averaged profile are then used to automatically count the number of bands present in the DAS data. The peaks are determined by two criteria: (1) only amplitudes above -13.8 dB (relative to maximum value) are chosen, and (2) there should be a minimum spacing of 54 s. The amplitude threshold was chosen to exclude the small peaks interpreted to originate from other acoustic events, whereas the spacing in time is introduced to avoid counting the same band twice (based on footage by NRK^27^). The total number of peaks counted is 87, one short of the number of bands reported by Oslo Municipality^25^.

The bands within the May 17 parade are clear in the data and can be successfully detected. It is, however, challenging to assess the exact number of individuals within each band from the data. This means that it is challenging to find the minimum detectable size for a crowd of pedestrians. The footage from NRK^27^ gives limited information about the exact number of individuals within each band but the relative size of the band can be extracted. Moreover, the footage is taken in front of the Royal Palace providing only indirect information about the different bands recorded on other fiber cable segments, e.g., in Sector 2 and 5. It is clear from the NRK footage and the DAS data that the strength of the generated signal depends on the size of the band and whether they walk in step or not. Using ample training data from additional data sources like surveillance cameras, manual observations, and/or airborne sensing the intensity of the data can be calibrated to quantify the specific size of the groups of pedestrians.

**Fig. 8:**
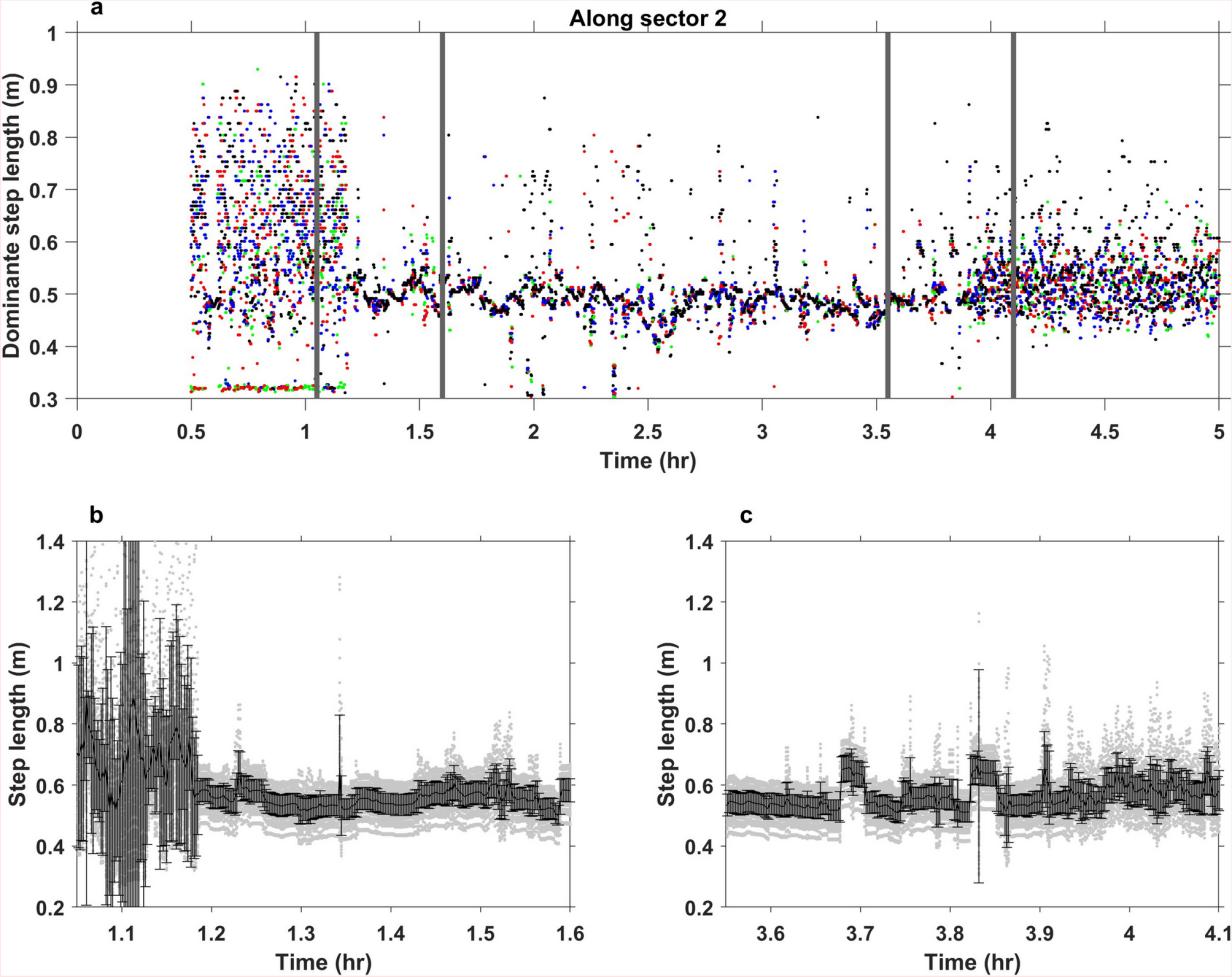
Dominant step lengths observed along Sector 2. (**a**) Step length estimates for a 4.5 hr period before, during and after the parade. (**b**) Zoomed display where the parade enters Sector 2. (**c**) Zoomed display when the parade dissolves. Display (**b**, **c**) include the uncertainty indicated via 90% prediction bars and with gray dots for each of the Monte Carlo samples.

### The step-lengths of the May 17 parade

The step length of a group of pedestrians can be computed for any time (*t*) by $l(t)=v/f(t)$, if the group’s frequency characteristics (*f*(*t*)) and walking pace (*v*) can be extracted from the data. Over the duration of the May 17 parade, the step lengths were computed as a function of time by extracting the frequency of the fundamental mode every 6.56 s (the temporal resolution of the spectrogram) and using the average speed over the sector studied. Figure 8a depicts the step length computed from data recorded on channel 760 m from the IU and eleven adjacent channels. Before the parade appeared in the data (the 1.1 h mark) it was not possible to reliably quantify the step length. When the parade walks above the channels the step lengths were concisely estimated between 0.44 m and 0.62 m, with a pattern changing as a function of the band walking above the fiber. After the parade passed the fiber segment (the 3.9 h mark), the spread in step length estimates rapidly grew.

To quantify the uncertainties in the step length estimates, the errors in the calculated velocities and frequencies are propagated using Monte Carlo sampling. Walking pace uncertainty is represented via the standard error in the regression analysis, whereas frequency uncertainties are computed by investigating the variation in frequency components between the eleven adjacent DAS channels, for each time sample. Step lengths are then computed for each time sample by drawing 100 independent samples of velocity and frequency. Figure 8b, c show the resulting step length uncertainties for the start of the parade (Fig. 8b) and the end of the parade (Fig. 8c). It is clear that step lengths cannot be resolved before the 1.1 h mark, whereas they can be resolved for at least one hour after the parade passes the segment, the 3.9 h mark. The uncertainty is lowest while the parade is above the segment.

The difference in step length within the parade is attributed to the number of people walking above the fiber at the recording time, and if the walk is synchronized. As expected, the step length computed at one channel varies as different bands pass above it. This can be observed as a sinusoidal-like oscillation of the modes in the spectrograms (Fig. 5) and the computed step lengths (Fig. 8a). The high amplitude modes in the spectrograms are attributed to being generated by the bands in front of the schools as these walk in step and thus transmit more energy into the subsurface. The sinusoidal-like behavior is interpreted to be due to the size of the bands and the age/height of the participants within the bands. An alternative explanation is that the oscillation is generated by the school(s) walking between the bands. The higher amplitudes are in this scenario attributed to the number of individuals between the bands, while the sinusoidal-like oscillation is attributed to how the schools are organized. For each school, the youngest pupils walk in the front and the oldest in the back. The footage from NRK^27^ supports the former interpretation in that the time between the bands corresponds to the timing of the modes in Fig. 5. Moreover, the majority of the bands walk in step which generates the observed over-harmonic modes (Fig. 6). To confirm whether the signals are generated by bands or schools a systematic manual inspection of the parade while it moves across the sectors containing fiber is required.

## Conclusion

In this work, an application of urban activity monitoring using DAS technology on existing fiber-optical infrastructure in the city of Oslo has been demonstrated. In particular, the dataset captures and characterizes moving crowds within the Norwegian National Day parade of 2023 (May 17).

By using DAS data, various portions of the May 17 parade were characterized by analyzing their frequency characteristics, walking speed, and step length. Wind bands march in step, which made it possible to pinpoint their locations in the parade. In the data along Sector 2 (Karl Johan street), automatic detection and counting successfully resolved 87 out of the 88 wind bands participating in the parade and estimated their step lengths to vary between 0.44 to 0.62 m. Additionally, it was possible to track the individual bands over two sectors (Karl Johan street and Haakon VIIs street) separated by around 1.5 km using crosscorrelation. A band before the main parade served as an initial test as it walked alone and identification was easy on both streets. Tracking within the main parade was more challenging as many bands with similar walking frequencies were walking near each other. For the initial street, clear and distinct tracks were obtained. For the second street, however, it was more difficult to separate the tracked band from the adjacent bands, due to the similar walk frequency and losing the signal for 1.5 km.

Note that the velocity varied along the parade path and for the different bands. A more detailed velocity analysis could be executed by computing the velocity as a function of time. Other interesting future work include more nuanced velocity estimation, tracking and crosscorrelation of the entire parade over time and space. Using ideas from dynamic time warping^28,29^, one could extend our approach of crosscorrelation to find the best alignment of DAS signals at different channels. This would provide a time-varying estimate of velocities for different parts of the parade.

Other studies have observed individual pedestrians in their DAS data. Rørstadbotnen et al.^30^ observed a pedestrian walking along a fiber dedicated to environmental monitoring. Furthermore, Jakkampudi et al.^15^ detect and characterize footsteps in a pedestrian-only area of the Pennsylvania State University and Wang et al.^17^ could see tracks from various bands in the Rose Parade directly in the time domain. The main challenge in carrying out a survey using a dark fiber as part of an existing network is that the coupling of the fiber optic cable might be unknown. Poor coupling will decrease the SNR of the affected channels. Additionally, the burial depth of the fibers in such a network might be unknown. This will affect the quality of well-coupled channels recording signals generated at the surface. The effect of unknown coupling and burial depth might limit large-scale monitoring of urban environments because portions of the fiber route might have low SNR levels. This decreases the detectability of small amplitude signals and hinders optimal conditions for DAS data analysis. In this study, tracks were achieved for large groups of people, like the parade on May 17, using an existing fiber optic cable network, but individual pedestrians were not observed. To obtain better knowledge of the position of the fiber cable and the coupling, a dedicated fiber should be installed close to the surface to observe smaller groups of pedestrians and improve the detectability of other acoustic events. This will incur additional costs.

To achieve the presented results, standard statistical and signal-processing tools were used to explore and understand the new urban dataset. There is room for improvement in classification and prediction methods by employing machine learning methodologies. There are for instance promising recent attempts of microseismic event detection from DAS data using convolutional neural networks^31^ and on traffic signal extraction from DAS using autoencoders^32^. Spatial-temporal correlation of the fiber relative to map coordinates is likely to enhance future real-time tracking of the events in an urban area.

DAS is one of the methods that can be used to monitor the activity in city areas using already deployed telecommunication networks. Since the capacity for one instrumentation exceeds 100 km, with a high spatial sampling, the interrogated fiber can cover vast areas of interest in a city. DAS measurements can be used to monitor the density and frequency of moving transportation vehicles, noise from buildings, mobility of people, and even unforeseen events. The use of available dark fibers will find additional applications that can be useful to the authorities managing urban areas. Municipal authorities would for instance like to know where crowds of different sizes are moving and then estimate the number of people to make general safety adjustments, if necessary. The information from DAS can be stored in a common database and combined with information from surveillance cameras, cell phone activity, and satellites. The use of DAS for such applications is still in its infancy, especially in urban areas. In the near future, development in interrogator engineering technology, signal processing, and data handling is expected. This in addition to the combined analysis of DAS data with other datasets will provide valuable information to decision support systems.

## Supplementary Information

Supplementary Information.

## Data Availability

Footage from the parade provided by Norwegian Broadcasting Corporation (NRK) is available through^27^. The dataset analysed for this study is available at DataverseNO via https://doi.org/10.18710/LBLYQT.
