# Supplementary material for: Monitoring bands during the Norwegian national day parade: a case study on urban distributed acoustic sensing
Source: Sci Rep. 2025 Apr 20;15:13629. doi: 10.1038/s41598-025-97017-z (PMC12009963; doi:10.1038/s41598-025-97017-z)
Supplement: Supplementary file 1 — Supplementary Information. [file 41598_2025_97017_MOESM1_ESM.pdf]

Monitoring Bands during the Norwegian National Day Parade: A Case Study on Urban Distributed Acoustic Sensing – Supplementary material

Robin Andre Rørstadbotnen[1,2,*], **Jo Eidsvik**[1,3], **Jan Langhammer**[4], **Martin Landrø**[1,2] **and Osman Mohammad Ibrahim**[5]
1: Norwegian University of Science and Technology (NTNU), Acoustic group, Department of Electronic Systems, NO-7491, O. S. Bragstads Plass 2A; Trondheim, Norway,
2: Norwegian University of Science and Technology (NTNU), Centre for Geophysical Forecasting, NO-7491, O. S. Bragstads Plass 2A, Trondheim, Norway;
3: Norwegian University of Science and Technology (NTNU), Department of Mathematical Sciences, NO-7491, Alfred Getz' vei 1, Trondheim, Norway;
4: Formerly: Norwegian University of Science and Technology (NTNU), Acoustic group, Department of Electronic Systems, NO-7491, O. S. Bragstads Plass 2A, Trondheim, Norway. Presently: Sensnet Analytics AS, Borgundvegen 340, 6009 Aalesund, Norway;
5: Oslo municipal authorities, Olav Vs gate 4, 0037, Oslo, Norway;
*: Corresponding author: robin.a.rorstadbotnen@ntnu.no.

The supplementary material consists of three parts. Firstly, an array analysis is described focusing on spatial aliasing. Then, material and figures that support the crosscorrelation presented in Section 3.2. Finally, the signal-to-noise ratio discussed in Section 4.2 of the main text is presented.

S1 Array analysis

The f–k transform is a common tool used to check whether there is any spatial aliasing in array data. Figure S1 depicts such a plot for band -1 along sector 2. This band walks alone so extracting its signal is trivial. A total of five modes are clear in the data. The first two are observed with a distinct frequency over all wavenumbers, while modes three through five are increasingly less energetic on higher frequencies and wavenumbers. All oblique lines are mapped into the same quadrant, indicating that there is no spatial aliasing present.

S2 The crosscorrelation procedure

This section addresses the suggested approach of crosscorrelation for tracking the parade using the frequency domain representation of the DAS data. It is presented in the following steps:

1. Find the reference frequency profile. This is done by computing the spectrogram of the channel with the best signal quality. The quality is assessed visually, but should be made automatic in future work. The spectrogram is then plotted and the crowd of people (in our case a band) is identified. Figure S2 shows an example spectrogram and the dashed line within depicts the crowd that is identified.
2. Start the outer loop going through all channels.
3. For the current channel in the loop, compute the spectrogram and extract the frequency range of interest. In this work we used frequencies between 1 and 10 Hz including the first four modes.
4. Start the inner loop, which loops through all time steps in the spectrogram.
5. For each time sample, crosscorrelate the corresponding frequency profile with the reference frequency profile and save the crosscorrelation coefficients.
6. Extract the crosscorrelation coefficients at zero lag and proceed to the next time sample.
7. When all time samples are done, proceed to the next channel.
8. The final result is a 2-dimensional matrix containing containing zero lag crosscorrealtion profiles for each channel.

In Figure S3 the autocorrelation that serve as the starting point for the tracking is shown. It works as a starting point due to the maximum correlation coefficient being unique and equal to 1. Figure S4a on the other hand show a typical zero lag output from a crosscorrelation, where the correlation coefficient is not euqal to 1. In this figure the maximum crosscorrelation coefficient is identified and the accompanying frequency profile is shown in Figure S4b. By repeating this for all channels a track can be made. Note that there are several coefficient above 0.9 and the correct track is made by starting at the autocorrelation maximum and identifying the channels, with high enough crosscorrelation coefficients, that connects to it. This is shown in Figure S5.

S3 Signal-to-Noise Ratio (SNR)

To quantity the improvements in Signal-to-Noise Ratio (SNR) through stacking, the ratio before and after the stack have been compared. The SNR is computed by extracting the amplitude level from the peaks in the frequency profiles corresponding to different bands (or more generally crowds of people), providing the signal (S) value, while the noise (N) is computed by extracting the mean between 50 channels before the arrival of the band during a period assumed to contain only background noise. The SNR is given as:

$$\mathrm{SNR} = \frac{S}{N}. \tag{S3}$$

Stacking coherent signals improves the SNR. To quantify this improvement the ratio between the stacked signal SNR and the unstacked signal SNR is taken: $\mathrm{SNR}_{\mathrm{stacked}}/\mathrm{SNR}_{\mathrm{unstacked}}$.

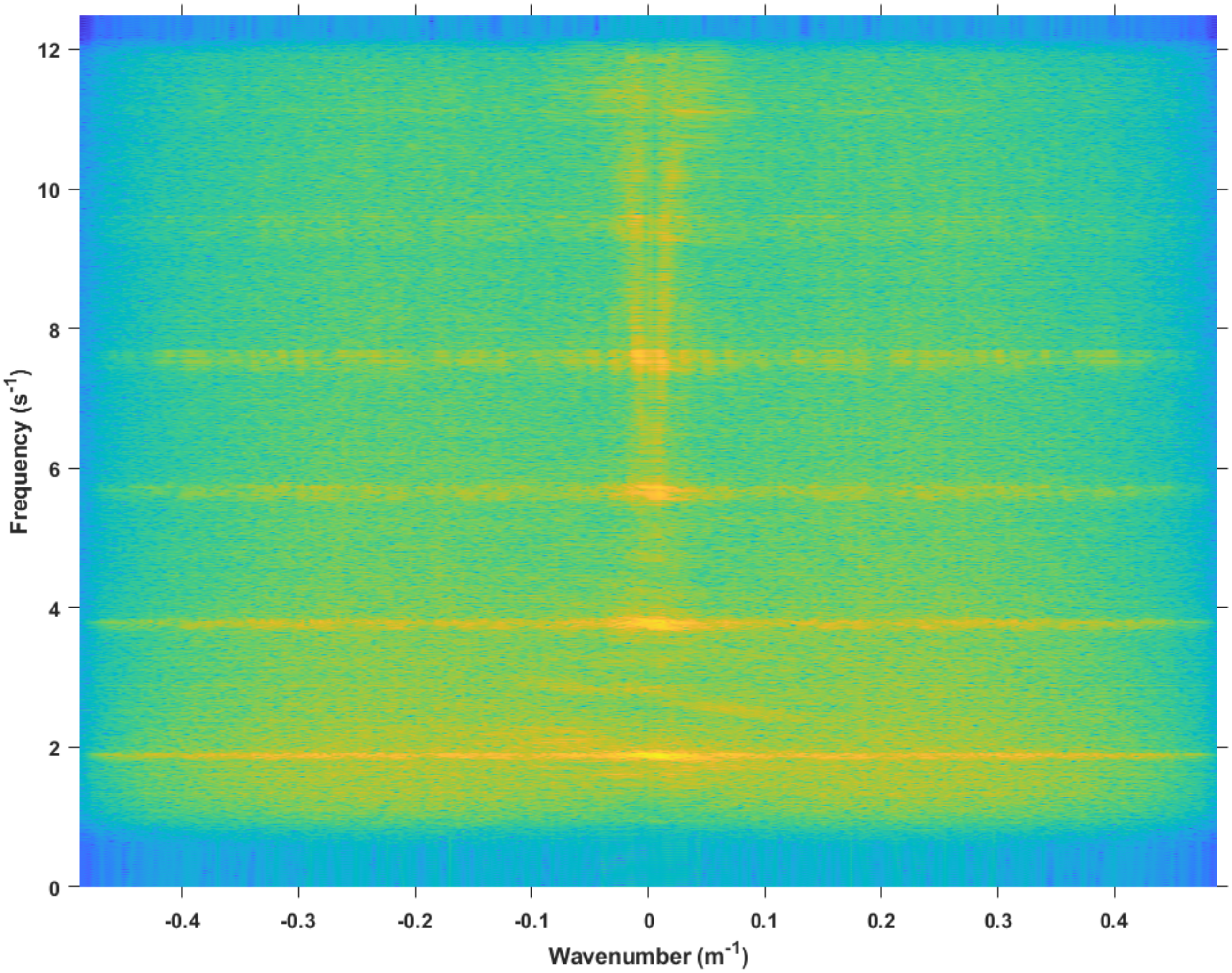

Figure S1. A f–k domain representation of band -1 moving along section 2 (see main text). No spatial aliasing is observed as the energy is consistently mapped into the same quadrants.

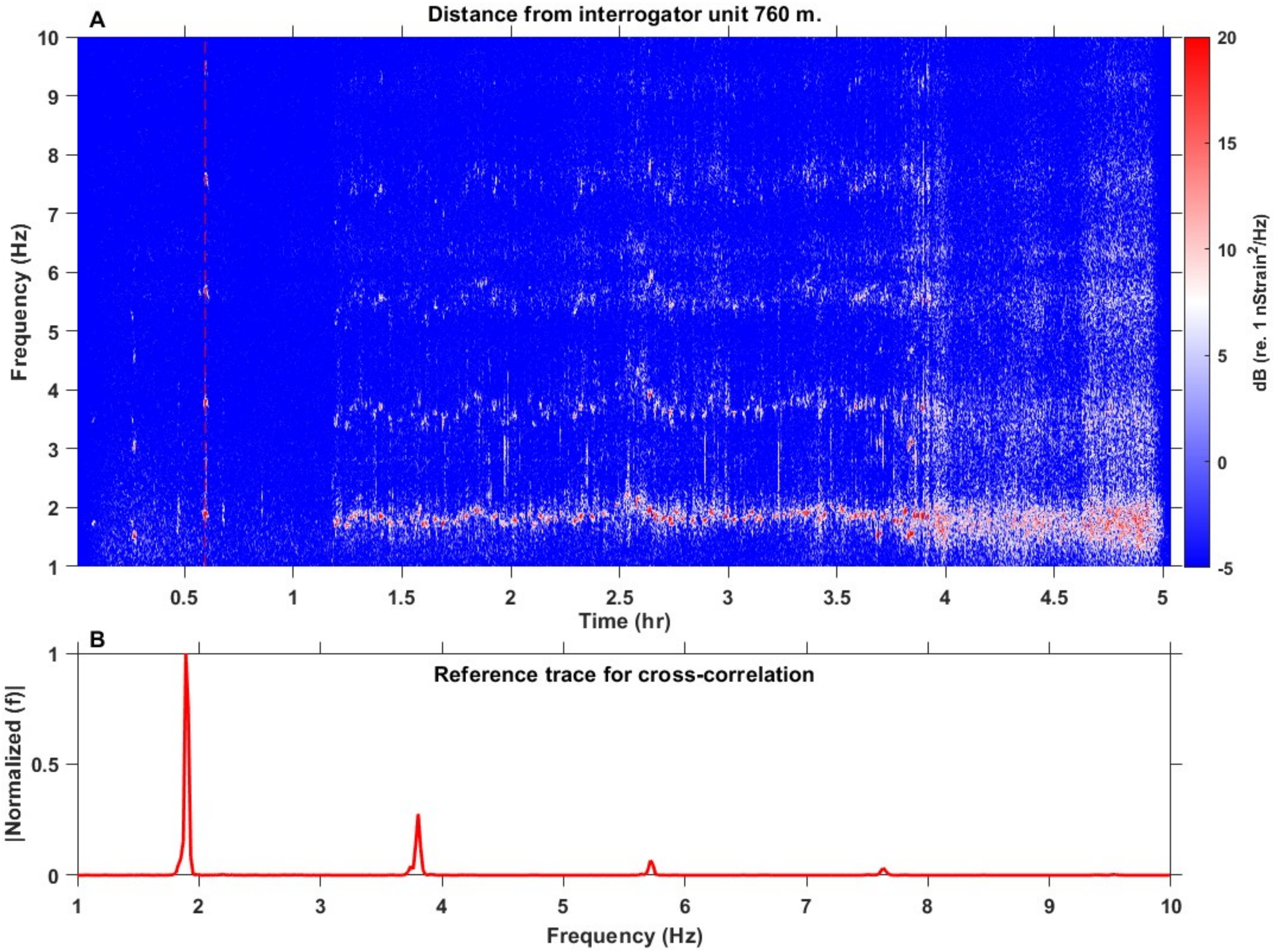

Figure S2. (a) Spectrogram for the reference channel. Red dashed line (at 0.6 h) indicate the profile extracted and shown in (b). The profile correspond to band -1 (see main text).

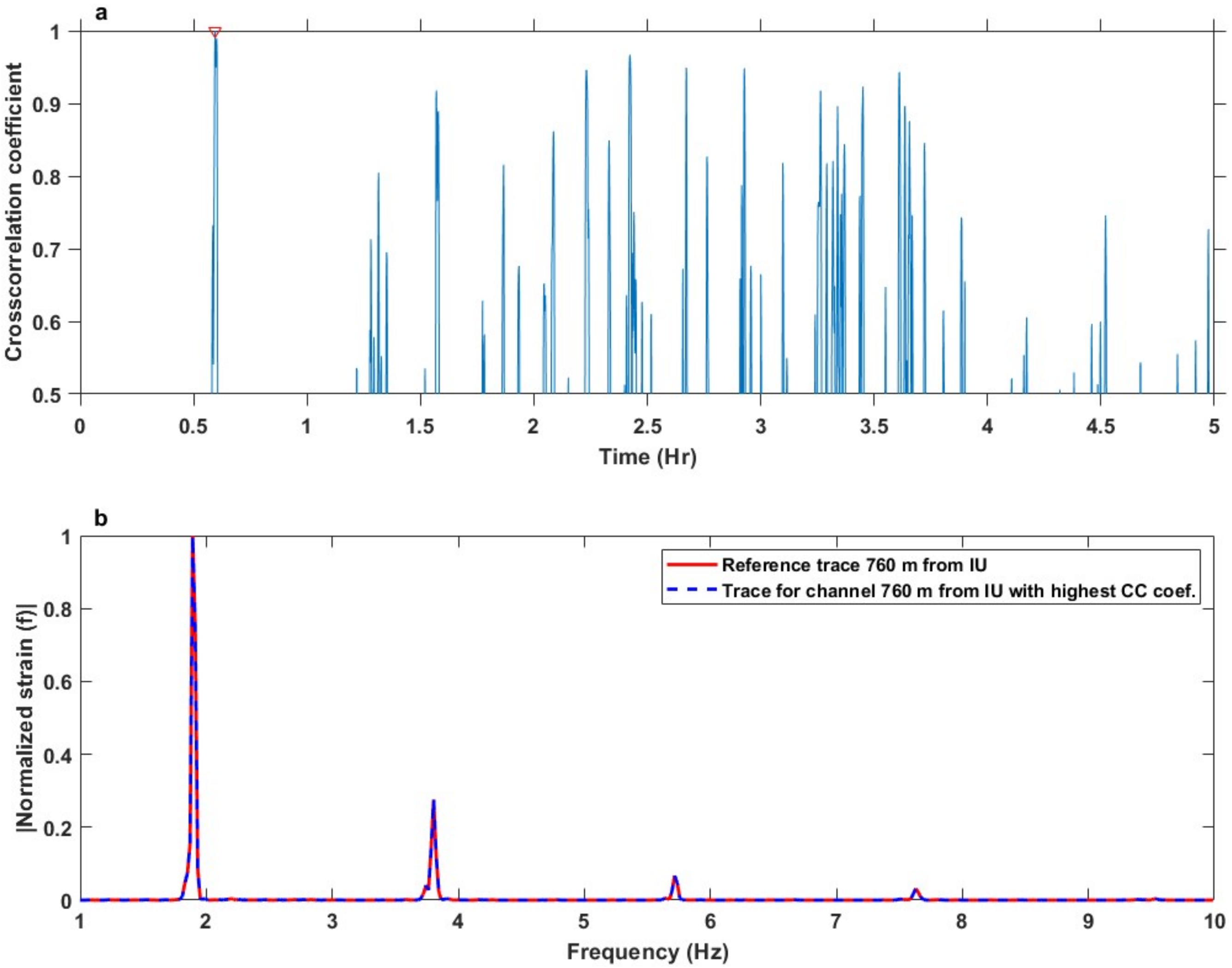

Figure S3. (a) Zero lag autocorrelation profile. The crosscorrelation value of 1 is correctly identified. Red triangle indicate the maximum coefficient. (b) The frequency profile of the reference trace (red) and the trace with the highest crosscorrelation coefficient.

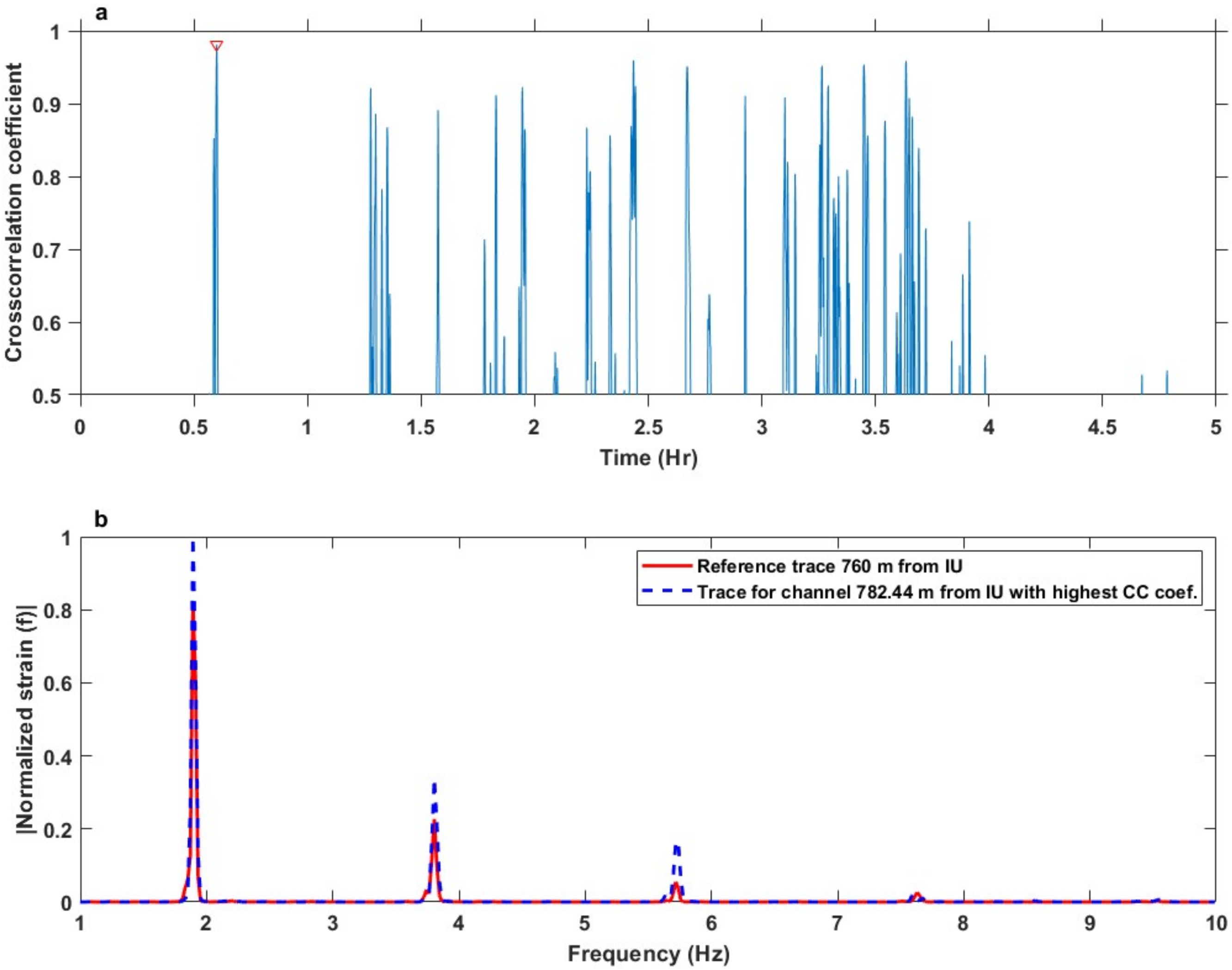

Figure S4. (a) Zero lag crosscorrelation profile for the reference channel and a channel 25.5 m further up the street. The maximum crosscorrelation has been shifted 20 s relative to Figure S3. Red triangle indicate the maximum coefficient. (b) The frequency profile of the reference trace (red) and the trace with the highest crosscorrelation coefficient.

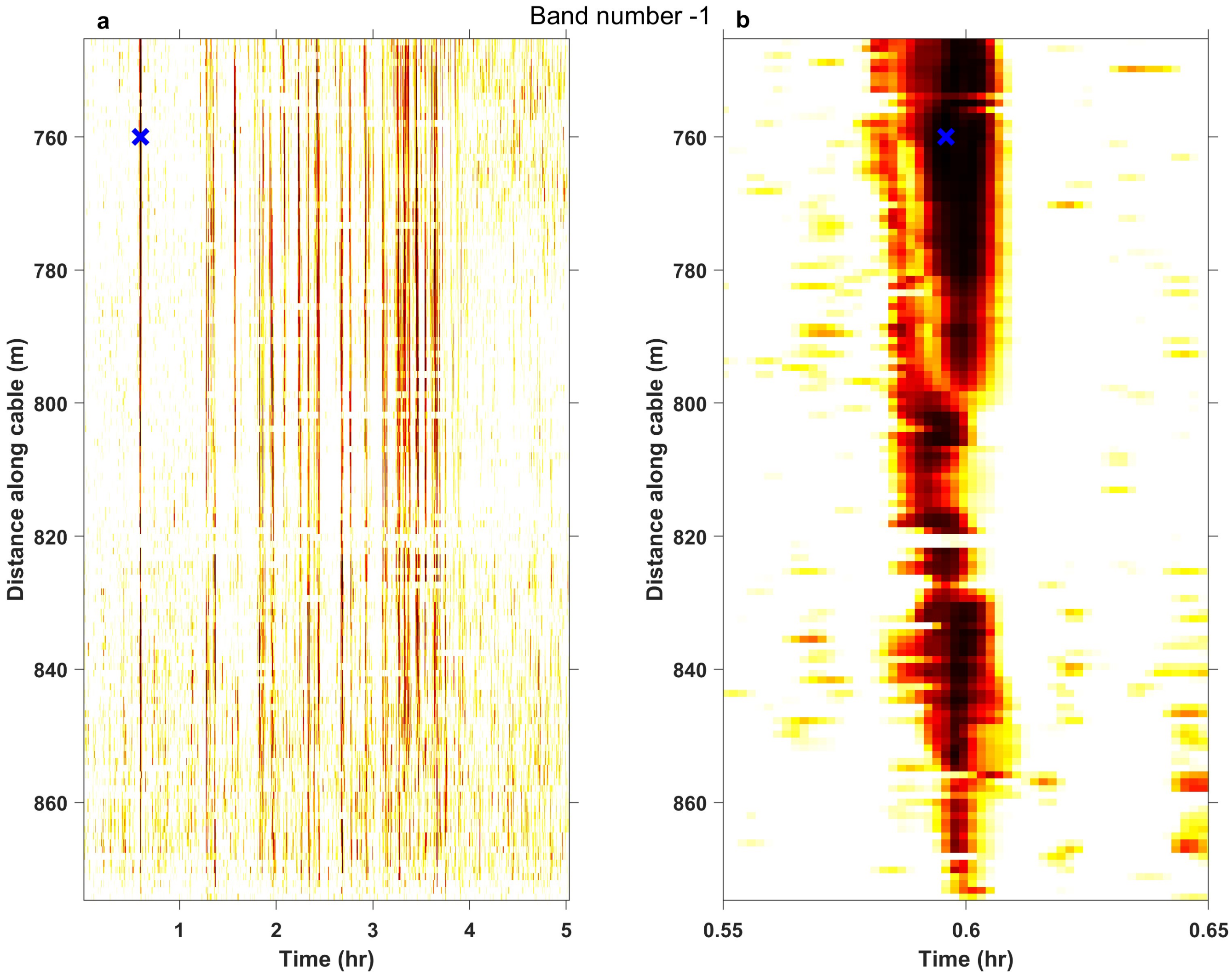

Figure S5. Band tracking using crosscorrelation. (a) Crosscorrelation of band -1 using a frequency profile between 1 and 10 Hz (containing modes 1 through 4). The channel at 760 m is correlated with the other channels along sector 2. (b) Zoomed in around the track of band -1.
